# Bone Tissue Engineering in a Perfusion Bioreactor Using Dexamethasone-Loaded Peptide Hydrogel

**DOI:** 10.3390/ma12060919

**Published:** 2019-03-19

**Authors:** Marina Panek, Maja Antunović, Lidija Pribolšan, Alan Ivković, Marijan Gotić, Andreja Vukasović, Katarina Caput Mihalić, Maja Pušić, Tanja Jurkin, Inga Marijanović

**Affiliations:** 1 Department of Biology, Faculty of Science, University of Zagreb, 10000 Zagreb, Croatia; marinapanek@gmail.com (M.P.); maja.antunovic@biol.pmf.hr (M.A.); lidija.pribolsan@gmail.com (L.P.); katarina.caput.mihalic@biol.pmf.hr (K.C.M.); maja.pusic@biol.pmf.hr (M.P.); 2 Center for Translational and Clinical Research, School of Medicine, University of Zagreb, 10000 Zagreb, Croatia; 3 Department of Histology and Embryology, School of Medicine, University of Zagreb, 10000 Zagreb, Croatia; alan.ivkovic@gmail.com (A.I.); andreja_vukasovic@yahoo.com (A.V.); 4 Department of Orthopaedic Surgery, University Hospital Sveti Duh, 10000 Zagreb, Croatia; 5 Department of Biotechnology, University of Rijeka, 51000 Rijeka, Croatia; 6 Department of Material Chemistry, Rudjer Boskovic Institute, 10000 Zagreb, Croatia; gotic@irb.hr (M.G.); Tanja.Jurkin@irb.hr (T.J.)

**Keywords:** hydrogel, RADA 16-I, dexamethasone, perfusion bioreactor, osteodifferentiation, human mesenchymal stem cells

## Abstract

The main goal of this study was the formation of bone tissue using dexamethasone (DEX)-loaded [COCH_3_]-RADARADARADARADA-[CONH_2_] (RADA 16-I) scaffold that has the ability to release optimal DEX concentration under perfusion force. Bone-marrow samples were collected from three patients during a hip arthroplasty. Human mesenchymal stem cells (hMSCs) were isolated and propagated in vitro in order to be seeded on scaffolds made of DEX-loaded RADA 16-I hydrogel in a perfusion bioreactor. DEX concentrations were as follows: 4 × 10^−3^, 4 × 10^−4^ and 4 × 10^−5^ M. After 21 days in a perfusion bioreactor, tissue was analyzed by scanning electron microscopy (SEM) and histology. Markers of osteogenic differentiation were quantified by real-time polymerase chain reaction (RT-PCR) and immunocytochemistry. Minerals were quantified and detected by the von Kossa method. In addition, DEX release from the scaffold in a perfusion bioreactor was assessed. The osteoblast differentiation was confirmed by the expression analysis of osteoblast-related genes (alkaline phosphatase (ALP), collagen I (COL1A1) and osteocalcin (OC). The hematoxylin/eosin staining confirmed the presence of cells and connective tissue, while SEM revealed morphological characteristics of cells, extracellular matrix and minerals—three main components of mature bone tissue. Immunocytochemical detection of collagen I is in concordance with given results, supporting the conclusion that scaffold with DEX concentration of 4 × 10^−4^ M has the optimal engineered tissue morphology. The best-engineered bone tissue is produced on scaffold loaded with 4 × 10^−4^ M DEX with a perfusion rate of 0.1 mL/min for 21 days. Differentiation of hMSCs on DEX-loaded RADA 16-I scaffold under perfusion force has a high potential for application in regenerative orthopedics.

## 1. Introduction

The world population is facing a significant increase in incidence of bone disorders and conditions. After the bone structure is disrupted, it can be restored completely without creation of fibrotic scar due to its unique biological properties. However, large bone defects caused by certain clinical situations cannot heal spontaneously and it is necessary to rely on bone regeneration strategies. Current techniques such as autologous and allogenic bone transplantation have their limits and disadvantages making space for improvement and development of new strategies. Bone tissue engineering emerged as a new approach to the treatment of large bone defects and might be able to solve the current limitations in their management. 

The bone tissue engineering process consists of three-dimensional (3D) bone graft production using autologous mesenchymal stem cells (MSCs), appropriate scaffold, osteogenic inducers as well as mechanical force in a bioreactor [1]. 3D-cell culturing mimics natural cell organization within tissues, but cell proliferation and differentiation can be decreased due to insufficient nutrient and oxygen transport, slow waste removal and non-uniform cell distribution. To overcome these limitations, systems that perfuse media through the scaffold can be used [2]. Perfusion bioreactors provide mixing of the media and a uniform distribution of cells enabling better environment control. Perfusion flow rate is a powerful tool that stimulates osteogenic differentiation because it applies mechanical stimulation to cells in the scaffold in the form of shear stress [3]. Therefore, a very important parameter is the flow rate that depends on the scaffold’s porosity, composition and geometry [4].

The ideal scaffold should be biocompatible, bioresorbable, osteoconductive, osteinductive, osteogenic and structurally similar to bone, as well as being user-friendly and cost-effective. Hydrogels, due to their unique biocompatibility, have long been used as matrices for tissue engineering. They can mimic the topography of extracellular matrix and can deliver bioactive molecules that promote bone regeneration [5]. A new generation of hydrogels is represented by self-assembling peptides, as they are completely biocompatible and biodegradable [6,7]. An ionic self-complementary peptide [COCH_3_]-RADARADARADARADA-[CONH_2_] (RADA 16-I) after induction forms a stable β-sheet structure and undergoes molecular self-assembly forming nanofibers and scaffold made of >99.5% of water [8]. RADA 16-I has been shown to support cell attachment as well as migration of various cell types [9,10], including osteoblasts and MSCs [11,12,13]. The additional advantage of RADA 16-I hydrogel is an efficient delivery of small molecules for sustained release applications [14,15]. 

In this study, we have used RADA 16-I peptide hydrogel for sustained release of dexamethasone (DEX) for bone tissue engineering in a 3D bioreactor system. DEX is a small steroid molecule that has been used for in vitro osteogenic differentiation. Usual DEX concentration in standard *in vitro* osteogenic culture protocols is 1 × 10^−7^ or 1 × 10^−8^ M over 21 days. DEX is applied together with ascorbic acid (Asc) and beta-glycerophosphate (β-Gly), a combination frequently called DAG. It stimulates cell growth and bone formation through activation of Runt-related transcription factor 2, a master transcription factor that is necessary for osteogenesis [16]. DEX has been used in human medicine for more than half of a century; hence, it is non-expensive, well characterized and easily accessible.

We hypothesize that MSCs can successfully differentiate and form bone tissue in the scaffold made of DEX-loaded hydrogel RADA 16-I in the specially adapted 3D perfusion bioreactor. Therefore, we loaded RADA 16-I hydrogel with DEX in concentrations of 4 × 10^−3^, 4 × 10^−4^ and 4 × 10^−5^ M to form a cell microenvironment in the bioreactor with a desired DEX concentration in the range from 1 × 10^−7^ to 1 × 10^−8^ M, similar to the physiological level of glucocorticoids. The specific aims of this research were the optimization of DEX concentration within the scaffold and in the microenvironment, measurement of the DEX release and evaluation of bone formation after 3D culture under the perfusion.

## 2. Materials and Methods 

### 2.1. Isolation and Expansion of Human Bone Marrow-Derived Mesenchymal Stem Cells 

The Ethics Committee of the former Traumatology Clinic (now Clinical Hospital Sestre Milosrdnice) approved the study (number 583/001, date of the approval 27.04.2010). The bone marrow samples were collected during a hip arthroplasty from three donors upon obtaining patients’ consent for participation in this research and publication of the data. The samples were taken at the former Traumatology Clinic (now Clinical Hospital Sestre Milosrdnice) in the time period between December 2010 and May 2013. Human mesenchymal stem cells (hMSCs) were isolated using a previously described method [17]. 

The cells were cultured in Dulbecco’s Modified Eagle Medium (DMEM)-low glucose (Lonza, Basel, Switzerland), supplemented with 10% fetal bovine serum (FBS) (Gibco Laboratories, Gaithersburg, MD, USA), 1% penicillin/streptomycin (Invitrogen, Carlsbad, CA, USA), 1% L-glutamine (Gibco) and 10 ng/mL fibroblast growth factor 2 (Cell Signaling Technology, Danvers, MA, USA). The cells were expanded in monolayer with media exchange every 2–3 days.

### 2.2. Peptide Hydrogels with Osteoinductive Signal

To compose scaffolds with an osteoinductive signal, RADA 16-I (Sigma-Aldrich, St. Louis, MO, USA) and DEX (Sigma-Aldrich) were mixed in a ratio of 9:1. Before mixing, DEX was dissolved in 100% ethanol at a concentration of 4 × 10^−2^ M. Scaffolds were prepared with the following DEX concentrations: 4 × 10^−3^, 4 × 10^−4^, 4 × 10^−5^ and 0 M (Table 1).

### 2.3. Three-Dimensional Cell Culture in a Perfusion Bioreactor

A perfusion bioreactor system (Cellec Biotek, Basel, Switzerland) was modified by adding two cell strainer meshes with 100 and 1 µm pores (BD Biosciences, Franklin Lakes, NJ, USA) to the adapter. Scaffolds were seeded with 2 × 10^6^ cells in a total volume of 10 mL media. Differentiation media was composed of Minimum Essential Medium Eagle-Alpha Modification (Lonza), 10% FBS, 1% penicillin/streptomycin, 1 mM sodium pyruvate (Sigma-Aldrich) and osteogenic differentiation inducers: 50 µg/mL Asc (Sigma-Aldrich) and 10 mM β-Gly (Sigma-Aldrich). Perfusion speed was set to 0.1 mL/min. RADA 16-I scaffold without DEX (control) was placed in the same media supplemented with DEX at 10^−7^ M. Media was exchanged every 2 to 3 days. After 24 h, media was collected and cell number was determined using a hemocytometer. After cell homing assessment, the DEX-loaded RADA 16-I scaffolds were tested in a bioreactor system for osteoblast differentiation. hMSCs were seeded onto scaffolds, exposed to differentiation inducers Asc and β-Gly for 21 days, and analyzed for bone markers.

### 2.4. Scanning Electron Microscope (SEM) Analysis of Tissue Sample and Dexamethasone Influence on Scaffold Morphology

RADA 16-I hydrogel forms a 3D peptide structure following exposure to salt solutions. Incorporation of other molecules can change the stability and the structure of the scaffold. To test whether incorporation of DEX (dissolved in ethanol) changes the structure, we observed RADA 16-I scaffolds with and without DEX using SEM. After 21 days in a perfusion bioreactor, tissue was analyzed by SEM. Tissue was fixed in 4% paraformaldehyde and washed in phosphate buffered saline (PBS) and tap water, dehydrated in ethanol (25%, 50%, 70%) and air-dried. The sample was deposited on the graphite foil glued to a carrier and mounted on SEM (JEOL USA Inc., Peabody, MA, USA). To create scaffold with DEX, 1% peptide hydrogel was mixed with 0.5 × 10^−3^ M DEX (1:1). The scaffold without DEX was prepared by mixing 1% peptide hydrogel with 50 mM calcium carbonate (CaCl_3_) (1:1).

### 2.5. Histological Analysis

Tissue was fixed, washed, dehydrated in ethanol dilutions, cleared in xylene and embedded in paraffin, Biowax blue (BioGnost, Zagreb, Croatia). Using a rotary microtome (Esselite Leitz, Stuttgart, Germany), tissue was cut into 4 and 5 µm thick sections. Sections were deparaffinized and stained with hematoxylin and eosin as well as with alizarin red solution (2%, pH 4.4) for 2 min. Following dehydration, slides were mounted with coverslips and observed by light microscopy (Olympus, Shinjuku, Tokyo, Japan).

### 2.6. Immunohistochemistry Staining

Immunohistochemistry staining was performed as previously described [18]. Antigen retrieval was performed using proteinase K (Agilent, Santa Clara, CA, USA) for 12 min, following immersion in 3% hydrogen peroxide (H_2_O_2_) solution and incubation in 10% goat serum (Agilent). The sections were then incubated with the anti-collagen I (Abcam, Cambridge, UK) diluted 1:400 in 1% goat serum in PBS, overnight at 4 °C. Real EnVision Detection System (Agilent) was used for visualization. Slides were counterstained with hematoxyline and observed by light microscopy (Olympus).

### 2.7. Isolation of Total Ribonucleic Acid and Gene Expression Analysis by Real-Time Polymerase Chain Reaction

Total RNA was isolated from cells using TRIzol reagent (Invitrogen, Carlsbad, CA, USA) according to manufacturer’s instructions. A total of 2 µg of total RNA was treated with DNAse I (Invitrogen) and reverse transcribed to complementary DNA (cDNA) using GeneAmp RNA PCR kit (Applied Biosystems, Foster City, CA, USA) [18]. RT-qPCR was performed using SYBR Green Mastermix (Applied Biosystems) and commercially available primers (Sigma-Aldrich, Table 2) on a 7500 Fast Real-Time PCR System (Applied Biosystems) under the following conditions: 10 min at 95 °C for 1 cycle, 15 s at 95 °C, and 1 min at 60 °C for 40 cycles. Relative quantification of the gene expression was performed using the 2^-∆∆Ct^ method.

### 2.8. Mineral Detection and Quantification

Precipitation of minerals was forced by increasing the perfusion to 0.3 mL/min. Media was collected with rinsed minerals, following detection by the von Kossa method. Quantification of deposited mineral was performed in media collected during the 21 days of culture. Media was centrifuged, and the pellet was dried by freeze-drying using a lyophilizer for 2.5 h. The mass of minerals was determined using analytical balance (Bosch, Stuttgart, Germany).

### 2.9. Dexamethasone Release from the Hydrogel Scaffold in a Perfusion Bioreactor

Since MSCs can differentiate only in specific range of DEX concentration, an extremely important step was the estimation of the DEX release tempo. Too high concentration would harm cells and too low concentration would fail to induce osteogenesis. To obtain DEX release kinetics, we set up a parallel system for the measurement of DEX release in the range of detectible levels of DEX using ultraviolet–visible spectroscopy (10^−5^ to 10^−4^ M). The scaffold containing 4 × 10^−3^ M DEX was placed in a bioreactor filled with 6 mL of PBS. Perfusion rate was set at 0.1 mL/min. Scaffold without DEX was used as a negative control. PBS was collected every 24 h for 16 days and the absorbance at 242 nm was measured using NanoVue. The concentration of DEX was calculated using the formula c(DEX) = A_242_/ε(DEX)xl.

### 2.10. Statistics

A one-way ANOVA analysis followed by Tukey’s multiple comparisons test of variance in GraphPad Prism 6.00 for Windows (GraphPad Software, La Jolla, CA, USA) was used for statistical analysis of the data. Results are presented as mean ± standard deviation (SD) with statistical significance set at *p* < 0.001. D’Agostino and Pearson omnibus normality test was used to determine the normality of data distribution.

## 3. Results

### 3.1. Dexamethasone Loading and Release

**Figure 1:**
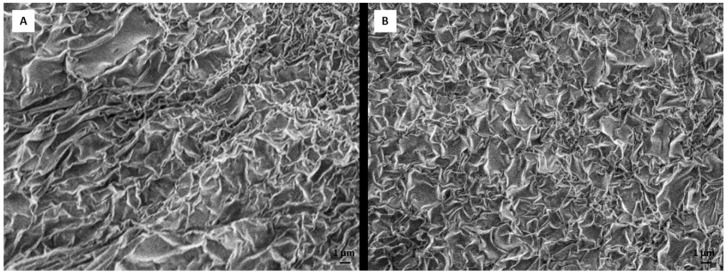
Scanning electron microscopy (SEM) images of 0.5% RADA 16-I peptide hydrogel 24 h after hydrogel polymerization, (**A**) without dexamethasone and (**B**) with dexamethasone. Scale bar represents 1 μm.

The structure of the hydrogel remained unchanged after incorporation of DEX, as can be seen in Figure 1. The difference in the polymerization time and stability due to DEX incorporation was not observed.

**Figure 2:**
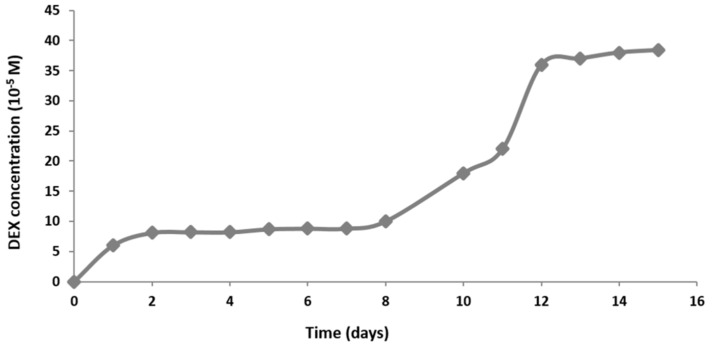
Concentration of DEX released to phosphate buffered saline (PBS) from RADA 16-I peptide hydrogel mixed with 4 × 10^−3^ M DEX in the parallel system of a perfusion bioreactor, measured each day for 15 days. Measurements were obtained by UV-Vis spectrophotometry.

Mimicking the natural bone environment in regard to physiological glucocorticoid levels, our target concentration of DEX in media was 10^−7^ M. The release of DEX was faster during the first two days, probably due to the surface rinsing of DEX. From day 2 to 7 the RADA 16-I scaffold held integrity and DEX release was very slow. After day 8, the degradation of RADA 16-I scaffold was progressing, resulting in accelerated DEX release. At day 14 the plateau in DEX release was reached (Figure 2).

From these results, we calculated the approximate values of concentrations that cells within the scaffold 4 × 10^−4^ M were exposed to, taking into account the simulation of media exchange points. Therefore, we estimated that over 14 days, cells were mostly exposed to DEX in the cell culture media in the concentration range of 10^−7^–10^−6^ M.

### 3.2. Cell Attachment and Differentiation

Data suggest that DEX incorporation negatively affects the number of cells attached to RADA 16-I scaffold. Scaffold without DEX has a cell-seeding rate of 98%, and the percentage decreases with increasing concentrations of DEX. Scaffolds with 4 × 10^−5^ and 4 × 10^−4^ M DEX have a cell-seeding rate of 88%, and scaffold with 4 × 10^−3^ M has a cell-seeding rate of 70%.

After scaffold analysis and cell homing assessment, the DEX-loaded RADA 16-I scaffolds were tested in a bioreactor system for osteoblast differentiation.

**Figure 3:**
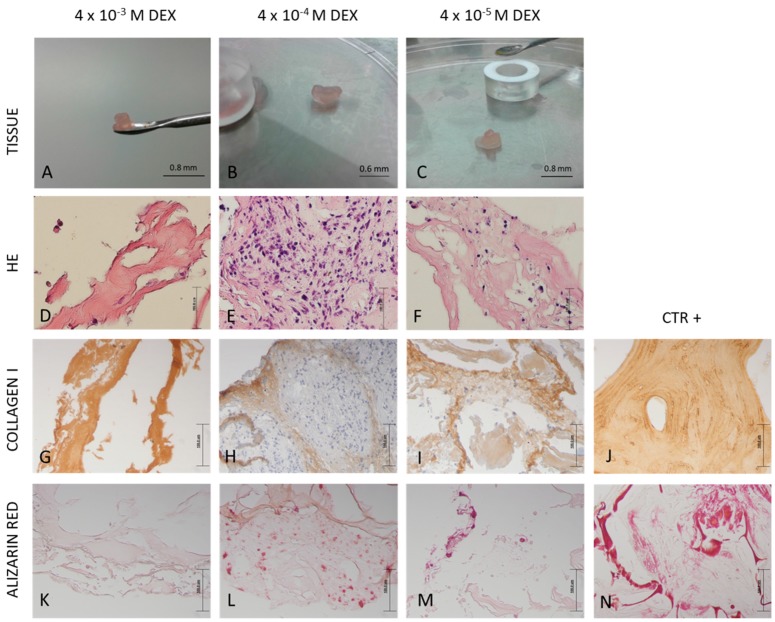
Macroscopic appearance and histology of in vitro engineered bone tissue in a three-dimensional perfusion bioreactor culture. RADA 16-I scaffolds were mixed with different concentrations of DEX: 4 × 10^−3^, 4 × 10^−4^ and 4 × 10^−5^ M, seeded with human mesenchymal stem cells (hMSC) and cultured for 21 days in a perfusion bioreactor system. Macroscopic appearance: (**A**–**C)**. Scale bar represents 0.8 and 0.6 mm. Histology appearance: (**D**–**F**) hematoxylin-eosin (HE) staining; (**G**–**J)** collagen type I immunohistochemistry staining; (**K**–**N**) Alizarin Red staining. Scale bar represents 100 μm. Positive control for immunohistochemistry staining is human bone. Positive control for Alizarin Red staining are hMSCs differentiated on the 30% hydroxyapatite scaffold for 21 days in a perfusion bioreactor.

Figure 3A–C presents tissue formed on RADA 16-I scaffold after 21 days of differentiation and it is evident that the presence of DEX in the RADA 16-I scaffold supports the stability of the engineered tissue. At day 21, the RADA 16-I scaffolds without DEX were mostly degraded and newly formed tissue was not strong enough to be analyzed by histology. Therefore, further analysis was performed only on DEX-loaded RADA 16-I scaffolds. To estimate the presence of the bone, engineered tissue was assessed by histology using hematoxylin/eosin staining and expression of collagen I was assessed by immunohistochemistry. RADA 16-I scaffold with 4 × 10^−3^ M DEX has significant amount of extracellular matrix surrounding very few cells. RADA 16-I scaffold with 4 × 10^−4^ M has the highest cell density and cells surrounded by good quality extracellular matrix. RADA 16-I scaffold with 4 × 10^−5^ M has a moderate number of cells and moderate matrix quality (Figure 3D–F). Collagen I expression is in concordance with histology results, supporting the conclusion that scaffold with a DEX concentration of 4 × 10^−4^ M has the optimal engineered tissue morphology (Figure 3G–J). Alizarin Red staining used for detection of calcium deposits in engineered tissue shows the best mineralization in the scaffold with DEX concentration of 4 × 10^−4^ M (Figure 3K–N).

**Figure 4:**
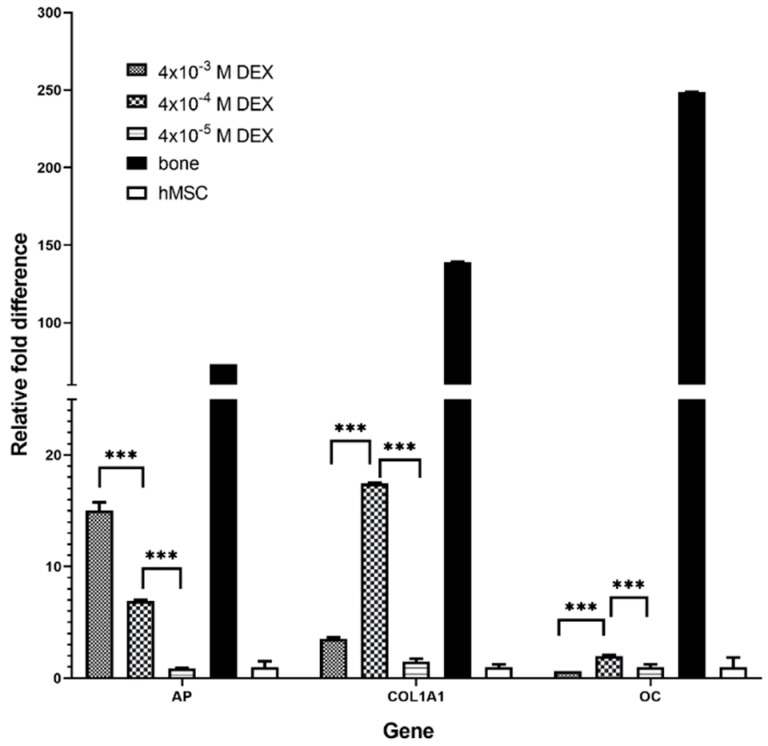
Relative gene expression of osteogenic markers in engineered bone tissue produced by cultivation of hMSCs on DEX loaded RADA 16-I scaffold with different DEX concentrations (4 × 10^−3^, 4 × 10^−4^ and 4 × 10^−5^ M) after 21 days in a perfusion bioreactor. Gene expression of alkaline phosphatase (ALP), collagen type I (COL1A1) and osteocalcin (OC) has been evaluated and normalized to the control (undifferentiated hMSCs). β-actin has been used as an endogenous reference and relative expression calculated using the ΔΔCt method. Data are shown as averages ± SD (*n* = 3). *** *p* < 0.001 indicates a significant difference between gene expression in produced engineered tissues, hMSCs and human bone samples.

Following histology and immunohistochemistry, messenger RNA (mRNA) expression of osteoblast-related genes such as alkaline phosphatase (ALP), collagen I (COL1A1) and osteocalcin (OC) in engineered tissue, human bone and undifferentiated hMSCs was analyzed. Among engineered tissue, when observing expression of COL1A and OC, the best differentiation is found in the scaffold with the concentration of DEX 4 × 10^−4^ M. With lower concentration of DEX in the scaffold (4 × 10^−5^ M), the mRNA levels of all analyzed osteoblast markers were lower (Figure 4). In the scaffold with the highest concentration of DEX (4 × 10^−3^ M), only ALP had higher expression. Therefore, the optimal DEX concentration among three tested concentrations of DEX (4 × 10^−3^, 4 × 10^−4^ and 4 × 10^−5^ M) in scaffold for osteogenesis and bone tissue applications would be 4 × 10^−4^ M according to COL1A1 and OC expression.

**Figure 5:**
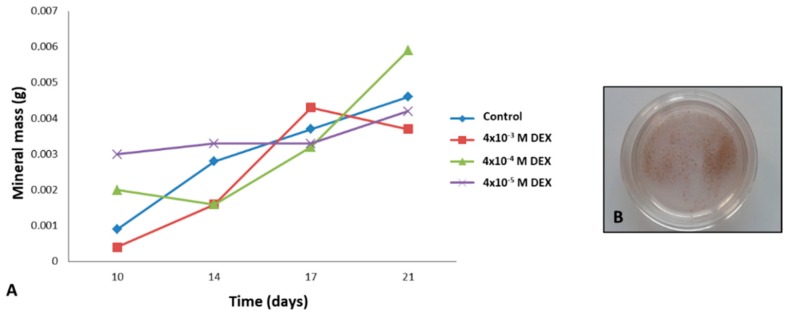
(**A**) Mass of deposited minerals in grams released from DEX loaded RADA 16-I scaffolds to media collected during the 21 days of culture in a perfusion bioreactor. (**B**) Presence of minerals in collected media confirmed with von Kossa staining.

As mineralization is also a valuable marker of bone tissue formation, we evaluated the quantity of minerals in scaffold (Figure 5A). Von Kossa staining confirmed that the collected material is composed of minerals (Figure 5B). From the data, it can be concluded that RADA 16-I scaffold with 10^−4^ M DEX produces the highest amount of minerals during 21 days in the bioreactor. These results were confirmed with Alizarin Red staining of engineered tissue (Figure 3K–N).

**Figure 6:**
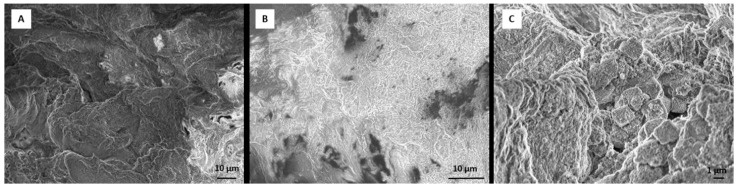
SEM images of engineered tissue on DEX-loaded RADA 16-I scaffold after 21 days of cultivation. Three morphologies that have characteristics of (**A**) cells; (**B**) extracellular matrix/connective tissue and (**C**) minerals can be observed. Scale bar (**A**,**B**) = 10 μm. Scale bar (**C**) = 1 μm.

Finally, SEM revealed that engineered tissue on DEX-loaded RADA 16-I scaffold contains morphological properties of all three components of bone tissue: cells (Figure 6A), extracellular matrix/connective tissue (Figure 6B) and minerals (Figure 6C).

## 4. Discussion

The main finding of this study is that peptide hydrogel RADA 16-I can be successfully supplemented with osteogenic activator DEX. The 10^−4^ M concentration of DEX within the scaffold is optimal for bone tissue engineering in a perfusion bioreactor. Therefore, our hypothesis that MSCs can successfully differentiate and form bone tissue in the scaffold made of DEX-loaded hydrogel RADA 16-I in the specially adapted 3D perfusion bioreactor, has been affirmed.

Perfusion bioreactors are mainly intended for use with solid porous materials and some adaptations were needed for the RADA 16-I hydrogel use. A filter mesh was used as a scaffold support as well as lower perfusion rate of 0.1 mL/min. Even though such low flow rates have been reported to increase cell viability, they do not seem to be optimal flow rates for osteoblast differentiation [19]. Wide variations of flow rates have been tested and optimal flow rates for bone tissue engineering applications seem to fall within the range of 0.2 to 1 mL/min [2]. Even though we used the flow rate below the recommended one, bone tissue formation was successful. However, clinical use of this system would not be possible without manufacturing modifications.

The most commonly used materials for bone tissue engineering are naturally occurring materials like chitosan, collagen, hyaluronic acid, alginate, elastin, cellulose, fibrin or gelatin. Some of those materials also have hydrogel properties, but a very important advantage of self-assembling RADA 16-I hydrogel are non-cytotoxicity and the ability of sustained release of bioactive molecules. Polymerization of RADA 16-I hydrogel is induced with any saline solution, while some other hydrogel drug release systems like chitosan and gelatin require glutaraldehyde for polymerization, causing low cytotoxicity. As a small, steroid molecule, DEX has a structure-defined problem to be covalently bound to naturally occurring scaffold components. Therefore, a better solution for DEX is to be entrapped in the free form within hydrogel pores. As the hydrogel degrades slowly, the DEX is being released inducing tissue formation that substitutes scaffold. The most important disadvantage of the RADA 16-I hydrogel is the softness of the material that is mechanically quite different from bone tissue. However, self-assembling peptides have been used to engineer bone [20] and cartilage [21] as well as other soft tissues [22]. RADA 16-I has been successfully used for bone tissue formation using rodent MSCs [23]. Our results confirmed that RADA 16-I scaffold can give enough support to human MSCs to produce extracellular matrix that subsequently mineralizes resulting in the formation of mature bone tissue. However, the size of the produced graft is limited, and weak mechanical properties limit the applications.

The successful bone formation is probably due to sustained release of DEX that was incorporated in RADA 16-I hydrogel. DEX is a small steroid molecule, capable of inducing bone-specific cellular response at the molecular level. We tested three different concentrations of DEX within the scaffold (4 × 10^−3^, 4 × 10^−4^ and 4 × 10^−5^ M) and the best engineered tissue was obtained with the 4 × 10^−4^ M concentration. In spite the highest expression of alkaline phosphatase in scaffold with 4 × 10^−3^ M DEX, the tissue morphology is inadequate, probably due to DEX cytotoxicity and/or lower cell attachment rate. However, the 10^−5^ M concentration of DEX in the scaffold was not sufficient to induce adequate osteogenic differentiation. When stem cells from human periodontal ligament were exposed to different concentrations of DEX, it has been shown that increase in concentration reduced cellular viability, with an increase in differentiation. Moreover, it has been shown that the concentration of 10^−7^ M promoted the most vigorous differentiation and mineralization [24]. *AP* is the marker of the early phase of osteogenic differentiation. Even though the presence of *AP* is important for osteoblast phenotype, the highest level of *AP* expression in the late phase (21 day) is not the measure of best osteoblast differentiation. Our gene expression data for COL1A1 and OC and histology (HE, Alizarin Red) suggest that the optimal DEX concentration within scaffold is 4 × 10^−4^ M.

The DEX release curve suggests that DEX release goes through three different phases. The first phase is characterized by fast release due to the rinsing of the untrapped molecules. The second phase is phase of slow release is due to slow degradation rate of hydrogel. During the last phase, the hydrogel is degrading rapidly, and quickly releases DEX residues. Studies of bioactive signal release from hydrogels in vitro have shown that hydrogels with higher peptide concentrations have a longer degradation period and retains the molecules trapped within for longer. As the hydrogel begins to break down due to hydrolysis and proteolytic degradation, molecules can move freely from matrix pores. In the beginning, the molecules from the hydrogel surface are released and then those from inside. Hydrogel pores are often larger than signal molecules and hydrogel releases most of the molecules within the first few days [25]. On the other hand, hydrogels with nanopores (like RADA 16-I) ensure increased molecule retention and their release depends entirely on peptide degradation. Since this is bioactive scaffold, it slowly releases DEX forming a cell microenvironment with a concentration in the range from 1 × 10^−7^ to 1 × 10^−6^ M. These concentrations are similar to the physiological level of glucocorticoids that play an important role in the normal regulation of bone remodeling [26].

DEX has been used as a differentiation inducer for the most of differentiation experiments in in vitro culture. It also has an approval for clinical use for other clinical applications, mostly as an anti-inflammatory drug. Other molecules that are finding their way to clinical applications are mostly recombinant proteins like BMP2 or BMP6, but they are more expensive and have their own side effects. Systemic and long-term use of DEX in patients does not seem to be good for bone, but we hypothesize that bone healing can benefit from local short-term use in the form of DEX-releasing scaffold.

## 5. Conclusions

In conclusion, our three-modality approach using perfusion in combination with a RADA 16-I scaffold and DEX sustained release can be useful for bone reconstruction, restoration and regeneration, as well as tissue engineering. hMSCs can successfully differentiate and form bone tissue in this setting, consisting of a RADA 16-I scaffold loaded with a DEX concentration of 4 × 10^−4^ M. Due to specific mechanical properties, this three-modality approach has high potential for specific bone healing applications like filling holes made by cysts, tumors, cavities in teeth or in any other situation where the location of an injury can be protected from mechanical loading during the healing process.

## Figures and Tables

**Table 1:** Dexamethasone (DEX)-loaded [COCH_3_]-RADARADARADARADA-[CONH_2_] (RADA 16-I) scaffold with three different concentrations of dexamethasone.

Scaffolds	Hydrogel Volume (µL)	Dexamethasone Volume (µL)	Dexamethasone Concentration (M)/Start	Dexamethasone Concentration (M)/Final
Scaffold I	135	15	4 × 10^−2^	4 × 10^−3^
Scaffold II	135	15	4 × 10^−3^	4 × 10^−4^
Scaffold III	135	15	4 × 10^−4^	4 × 10^−5^
Control	150	0	0	0

**Table 2:** Human primer sequences used for determination of relative gene expression levels by reverse transcription-polymerase chain reaction analysis.

Genes	Sequence 5′–3′	Tm (°C)
COL1A1	Forward GCTATGATGAGAAATCAACCG Reverse TCATCTCCATTTCCAGG	61.1 61.6
OC	Forward TTCTTTCCTCTTCCCCTTG Reverse CCTCTTCTGGAGTTTATTTGG	60.8 59.3
ALP	Forward CTGTTTACATTTGGATAC Reverse ATGGAGACATTCTCGTTC	57.4 61.6
